# Supplementary material for: Acceptability of breast milk donor banking: A qualitative study among health workers in Greater Accra Regional Hospital, Ghana
Source: PLOS Glob Public Health. 2023 Aug 16;3(8):e0001870. doi: 10.1371/journal.pgph.0001870 (PMC10431602; doi:10.1371/journal.pgph.0001870)
Supplement: Attachment — Submitted filename: Response to Reviewer.docx [file pgph.0001870.s001.docx]

**Response to Reviewers**

Breastmilk Banking

Reviewer #1: Congratulations on your paper! You have done a great work! It is essential to understand health workers knowledge about breast milk donor bank process before implementing this kind of health facility. Still, I have some comments to share:

**Introduction:** has relevant information and guides readers to comprehend your objectives.

**Response:** Thank you very much for review

**Methods**: concise, detailed, well structured. Recording interviews is a clever way to review information and clarify any misunderstanding.

**Response:** Thank you very much for review

**Results:** Presenting your findings in sections makes it easier for readers to analyze them.
Data presented in Table 1 diverges from data in text.

**Response:** Thank you very much for review

Discussion: your results corroborates the findings presented in other articles.
You should consider making your data available through an easier access link, such as a support information link.

**Response:** Thank you very much for review

Reviewer #2: Abstract
Concise and clear. p.2

**Response:** Thank you very much for review

Introduction
Clarify the sentence ʿIf possible and safe, WHO advises........., may be hamperedʾ. line 81 and 82 . p. 4

**Response:** The sentence has been revised. It now read “The WHO recommends replacement feeding when breastfeeding, especially exclusive breastfeeding is not possible for medical reasons. Kindly seepage 4 lines 81-83.

Reference the texts on line 92-97. p. 4

**Response:** This has been done. It is reference 13 on page 4

Methods
Study area
Put the following information ʽThe data for the study was collected among health workers at Greater Accra Regional Hospital. The hospital renders antenatal and delivery services to pregnant women and is a secondary level government hospital supporting the leading referral centre in Korle-Bu Teaching Hospital, Accra, thereby positioning it favourably as the study settingʼ under study site and reference it. line 115-119. Also include information on the total number of health workers in the facility P. 5

**Response:** We has been addressed and highlighted on page 5

Population
Correct the statement ʽAccra's Greater Accra Regional Hospitalʼ on line 114 P.6
Also include information on the total population of health workers in the facility

**Response:** This has been addressed and highlighted on page 11

Include the eligibility criteria

**Response:** The inclusion and exclusion criteria has been added on page 11

Data collection tool and procedure
How did you ensured reliability and validity of the interview guide?
What language was used to conduct the interviews? Which type of interview was conducted? P. 6
**Response:** These have been addressed and highlighted on pages 7 and 8.

Results
Sociodemographic characteristics
Clarify the statement ‘There were five nurses’, line 175 p. 8

**Response:** This refers to general nurses. This addition has been done.

Perception of health workers regarding breastmilk donor banking
Clarify the statement ʽBenefits of breastmilk donor bankingʼ on line 218 p.11. Is this a main theme or sub-theme? Is this the same as the significance of breastmilk?

**Response:** The theme as been revised to benefits of breastmilk donor banking

Can you clarify the sub-theme that line 264-282 is addressing? p.13 to 14
I suggest that the result be presented under the main theme. The cultural, ethics and religious considerations theme was presented under benefits of breastmilk in the manuscript. P. 14. This theme should be presented separately.

**Response:** These have revised on page 13.

Clarify the sentence some people believed that given a baby's immunization, it wasn't safe to use it to feed themʼ, line 299-300 p.14

**Response:** This sentence has been revised and highlighted on page 15

Line 421 to 430, Funding and sustainability were not captured as a sub-theme. I suggest you put it as a sub-theme under challenges and concern p.10

**Response:** These have been included as sub-theme as suggested on page 10

Correct the sentence ʽFrom the planning stage through the implementation stage, education was advised at every stageʼ as the intervention has not been carried out yet. line 481-482, p.22.

**Response:** This has been revised and highlighted on page 23.

The statement ʽinvolve stakeholders in the development of a policy..........ʼ on line 509-510 was not captured as a sub-theme under pre-implementation. p.23.

**Response:** This has been added as sub-theme

The result for the themes; opportunity cost and intervention coherence under acceptability were not reported.

**Response:** This has been added on page 21

Limitations
Report on the limitations of the study

**Response:** This has been added on page 30.

Discussion
Support line 539 -550 with similar or contrary research findings p.25.

**Response:** This has been addressed

Conclude each paragraph with implication or recommendation as you have done in paragraph 6 of the discussion. P.27

**Response:** These have been done
Provide references for the sentence ʽThis recommendation was in line with a number of studies......ʼ. Line 614-616, p.28

**Response:** These have been addressed

Include the main contribution of the study to your field of practice or academic knowledge.p. 28

**Response:** These have been added
