# Supplementary material for: Acceptability of breast milk donor banking: A qualitative study among health workers in Greater Accra Regional Hospital, Ghana
Source: PLOS Glob Public Health. 2023 Aug 16;3(8):e0001870. doi: 10.1371/journal.pgph.0001870 (PMC10431602; doi:10.1371/journal.pgph.0001870)
Supplement: Attachment — Submitted filename: Response to Journal Editorial Team.docx [file pgph.0001870.s002.docx]

**Response to Editorial Team**

Thank for very much for your comments Breastmilk Banking

Comment

1. Please review your reference list to ensure that it is complete and correct. If you have cited papers that have been retracted, please include the rationale for doing so in the manuscript text, or remove these references and replace them with relevant current references. Any changes to the reference list should be mentioned in the rebuttal letter that accompanies your revised manuscript. If you need to cite a retracted article, indicate the article’s retracted status in the References list and also include a citation and full reference for the retraction notice.

**Response:** We addressed all the comments on the reference list. We included the missing doi, PMID and pages numbers of the highlighted references on page 31-34 on our reference manager and refreshed to effect the update. No paper has been retracted from the list.
